# Monthly Summary

**Published:** 1879-02

**Authors:** 


### MONTHLY SUMMARY.

*Imbedding of a Piece of Tooth in the Tongue, in a Case of Gunshot Wound.*—Dr. John H. Packard writes to the *Medical and Surgical Reporter*, as follows:

In the *British Medical Journal* for September 21st, 1878, there is mentioned the following case, reported by Bronstein. A Russian soldier was wounded in the mouth at Plevna, July 18th, 1877. Nine months subsequently he felt pain in the tongue, which was found to contain a foreign body. On extraction this body proved to be an incisor tooth, carried away by the ball.

On reading this, I was reminded of a somewhat similar case which came under my notice during the late civil war. Major S., of the Sixth Pennsylvania Cavalry, was riding across the field on the 7th of May, 1864, during the battle of the Wilderness, when a ball entered his mouth, through the right cheek, breaking away the bicuspid and anterior molar teeth of the upper jaw on that side, cutting a gash across the tongue, and shattering the left upper molar teeth, with their alveoli. Although stunned by the blow, Major S. was conscious of spitting out a mouthful of blood and pieces of teeth, among which the ball must have been, for it was never seen afterward, and the left cheek was not perforated. In four days he reached his home, when I attended him. He thinks it was on the thirtieth day that he called my attention to a swelling and soreness on the upper surface of the tongue, where, he said, there had twice before been, at intervals of two or three days, a slight discharge of pus. Upon careful pressure I felt fluctuation, and opened a little abscess. Passing a fine probe through the orifice, I felt a foreign substance, which was at once extracted, and proved to be a piece of one of the teeth. It need hardly be added that the cavity healed up soundly at once.

---

*Sugar.*—On an average, every man, woman and child in the United States consumes each year about 30 pounds of cane sugar, and nearly 2 gallons of molasses, besides maple sugar, honey and other sweets. 19 lbs. of pure cane sugar is actually made up of, and can be changed into 8 lbs. of charcoal and 11 lbs. of water! Pure white starch is made up of 8 lbs. of charcoal (carbon) and 10 lbs of water. Any boy can demonstrate this roughly by putting a small quantity of sugar on a piece of thin iron over a hot lamp or coals, and hold over it a glass jar bottom up. The sugar will change to pure charcoal, while the water will rise up and condense on the inside of the jar, if it be kept cool, and he will get nothing from the sugar but coal and water. The chemist can easily take the 19 lbs. of sugar and change it into 8 lbs. of charcoal and 11 lbs. of pure water, though he has not yet learned how to put the coal and the elements of the water together to produce the sugar. That requires the action of the living plant. Our sugar comes mainly from the sugar cane grown in the Southern States (most from Louisiana,) and from the West India Islands. The canes are somewhat like cornstalks, but larger, taller, with narrower leaves. The sap or juice of the cane is pressed out between iron rollers, then boiled down to syrup, which crystallizes into sugar grains in large vats. Most of the sugar used in Europe is from the juice of the sugar-beet. It is similar to our cane sugar. The raw sugar is refined chiefly in Northern cities, by dissolving it, straining it through cloth, and through burned bones, after which it is boiled down until thick enough to crystallize in grains.—*American Agriculturist.*

---

*Anæsthetic for Children.*—With a large experience in the use of chloroform as an anæsthetic, Prof. Demme arrives at the conclusion to prefer it over all others as an anæsthetic for children. He says, its action is quicker, and more reliable, and in no way more dangerous than that of the others. In 32 cases he produced anæsthesia with ether and among those cases there were eight in which dangerous symptoms occurred, which made it necessary to employ energetic means to revive the little patients. D. complains, especially of the long duration of the stage of excitement, and of the severe emesis which frequently took place during, or after the administration of ether. He also mentions many instances, in which bronchitis, and a few in which disturbances of the bowels followed the use of ether. Bichloride of methylene had been employed in twenty-eight cases; the children took it better than either chloroform or ether, none of the unpleasant complications of ether narcosis were observed, but profound anæsthesia, such as is frequently required, could not be produced by it. The chloride of æthyliden—highly recommended as a safe anæsthetic by Dr. Liebrich—was used in twenty cases; while under its influence, a child of eighteen months, had a sudden and severe attack of asphyxia, which made it necessary to resort to the use of artificial respiration.—*Paul H. Kretzschmar,—Hospital Gazette.*

---

*Effect of Diet on Liquor Drinking.*—Charles Napier, an English scientific man, has been testing the truth of Liebig's theory that liquor drinking is compatible with animal food, but not with a farinaceous diet. The experiment was tried upon twenty-seven liquor drinking persons, with results substantiating the Liebig theory. Among the more striking instances of reform brought about by a change of diet was that of a gentleman of 60, who had been addicted to intemperate habits for thirty-five years, his outbursts averaging once a week. His constitution was so shattered that he had great difficulty in insuring his life. After an attack of delirium tremens, which nearly ended fatally, he was persuaded to enter upon a farinaceous diet, which, we are assured, cured him completely in seven months. He seems to have been very thin at the beginning of the experiment, but at the close of the period named had gained twenty-eight pounds, being then of about the normal weight for a person of his height. Among the articles of food which are specified by Napier as pre-eminent for antagonism to alcohol, are macaroni, haricot beans, dried peas and lentils, all of which should be well boiled and flavored with plenty of butter or olive oil. The various garden vegetables are said to be helpful, but a diet mainly composed of them would not resist the tendency to intemperance so effectually as one of macaroni and farinaceous food. From this point of view, high glutinous bread would be of great utility, but it should not be sour, such acidity being calculated to foster the habit of alcoholic drinking. A like remark may be applied to the use of salted food. If we inquire the cause of a vegetarian's alleged disinclination to alcoholic liquors, we find that the carbonaceous starch contained in the macaroni, beans or oleaginous aliment appears to render unnecessary, and therefore repulsive, carbon in an alcoholic form.—*N. Y. Graphic.*

---

*Camphor as a Hypnotic.*—Wittich has repeatedly administered camphor to relieve the insomnia which accompanies certain forms of mania, hysterical insanity, and hypochondria. He has found that, under such conditions, camphor acts much better than chloral, morphine, or bromide of potassium. He administers it by hypodermic injection. He dissolves it in olive oil, and the dose which he recommends is from one to one and one-quarter grain. Small doses are more certain to produce sleep than large doses The sedative effect, as a rule, appears rapidly and the sleep produced lasts several hours. The injection is to be repeated when the restlessness reappears.—*Jour. de Med. de Bordeaux.*

---

*Paper Teeth.*—Paper teeth are a new invention in Germany, and a number of specimens were displayed at the late paper exhibition in Berlin. They are warranted fully as durable as any other teeth.

*The Goodyear Dental Vulcanite Co.* have gained their suit against Adolphe P. Preterre, of New York. The decision rendered by Judge Wallace seems to place the whole matter of making and repairing rubber and celluloid plates under the control of the Cummings patent. The following are the principal points in the decision:

To repair a dental plate made according to the Cummings patent, by the substitution of a new for an old portion, with teeth imbedded, is an infringement of the patent.

It is an infringement of the patent if the teeth be embedded in rubber according to its terms, although the remaining portion of the base plate be made entirely of gold.

Celluloid and rose pearl held to be the substantial equivalent of the hard rubber or vulcanite in all essentials necessary to the successful practice of the invention, and their use a violation of the rights under the Cummings patent.

The facts established by the evidence in relation to such equivalency being different from those appearing in *Goodyear Dental Company vs. Davis, et al.*, that case is not deemed to be in point, although it declared the non-equivalent character of celluloid.—*Dental Advertiser.*

---

*Borax as an Antiseptic.*—At a recent meeting of the Academy of Sciences of Lombardy, G. Polli reported the results of numerous experiments in which beer, meat, eggs, blood, and urine were treated with boracic acid and borax for thirty days during the summer time, and were found still to retain their freshness and to present no traces of fermentation having taken place in them. In experiments, on the other hand, without the addition of the salt, but in some cases with the addition of sulphate of soda, the fluids passed into a state of complete decomposition in the course of fifteen days. The energetic disinfecting power possessed by boracic acid and borax, and the facility with which these substances can be absorbed into the economy, led Polli to recommend their use in diseases in regard to the infectious nature of which no doubt exists, or in which septic conditions readily arise. He adduces several examples in which the febrile conditions of tuberculosis under-went diminution. No benefit was obtained by Professor Visconti from experiments made with these remedies in malaria, though other observers have arrived at a different conclusion. In chronic cystitis the muco-purulent discharge quickly diminished, and even altogether disappeared in the course of a few days, and rapid improvement occured in cases of bad suppurating wounds when they were applied externally. The dose recommended by Polli is 75 grains of boracic acid and 150 grains of borax per diem.—*Druggists Circular.*

*Neuralgic Toothache.*—Dr. Darvaris recommends quinine powder as a local application for neuralgic toothache. The patient should dip a finger into fresh water and then into the quinine powder, and rub it forcibly on the gum in the neighborhood of the painful tooth. The application should be repeated two or three times in succession. The bitter taste of the medicine should be borne as long as possible. Dr. Darvaris tried this remedy first on himself, and then on numerous other persons, among them many who had tried other remedies in vain. It invariably produced a rapid alleviation of the pain.—*Allg. med. Cent. Zeit.*

---

*Chloral as a Revulsive.*—This is the subject of a paper in the *Bulletin de Therapeutique*, No. 94, by Dr. H. Peyraud. Made into a mass with gum tragacanth, spread on paper and applied to the skin, it will produce a blister without pain. Applied as a powder, on cotton, it causes a painful burning sensation. By the former method, a portion is absorbed and the patient falls asleep. Its action is not so uniform as cantharides, but as a mild vesicant, or an agreeable revulsive, the author quoted would commend such "chloral paper" to physicians, the more so as it will keep for months without losing its activity, if well prepared.—*Med. and Surg. Reporter.*

---

*Cuprum Ammoniatum in the Treatment of Neuralgia.*—M. Bourdon has for several years used with success the ammonia-sulphate of copper in the treatment of neuralgias that prove rebellious to the ordinary methods of cure. M. Ferreol, of the *Hospital Lariboisiere*, has lately had recourse to the same drug in two cases of facial neuralgia, in which hypodermic injections of morphine and aconitine, arsenical preparations, the nitrate of aconitine, and tincture of gelseminum had proved utterly useless. He administered it in doses at first of 1½ grains and then of 2¼ grains per diem. The relief obtained was so rapid that M. Ferreol felt himself constrained to publish his cases.—*Bulletin de Therapeutique.*

---

*Convulsions Caused by a Hair.*—A child under one year of age suffered for several weeks from convulsions, which varied in severity and were frequently repeated. It appeared to be healthy in all other respects. All the usual methods of treatment were employed without success. At last the mother noticed the end of a hair lodged between the two incisors of the child, and, on drawing on it, removed a hair nearly a yard in length, which had hung down into the throat of the little patient. After the removal of this foreign body, the convulsions ceased as if by enchantment.—*Druggists Circular.*

